# Consecutive Treatment with Brain-Derived Neurotrophic Factor and Electrical Stimulation Has a Protective Effect on Primary Auditory Neurons

**DOI:** 10.3390/brainsci10080559

**Published:** 2020-08-15

**Authors:** Verena Scheper, Ira Seidel-Effenberg, Thomas Lenarz, Timo Stöver, Gerrit Paasche

**Affiliations:** 1 Department of Otorhinolaryngology, Hannover Medical School, Carl-Neuberg-Str. 1, 30625 Hannover, Germany; iraeffenberg@aol.com (I.S.-E.); lenarz.thomas@mh-hannover.de (T.L.); timo.stoever@kgu.de (T.S.); paasche.gerrit@mh-hannover.de (G.P.); 2 Hearing4all Cluster of Excellence, Hannover Medical School, Carl-Neuberg-Str. 1, 30625 Hannover, Germany; 3 Department of Otorhinolaryngology, Johann Wolfgang Goethe-University, Theodor-Stern-Kai 7, 60590 Frankfurt am Main, Germany

**Keywords:** BDNF, ES, spiral ganglion cells, cochlear implant, inner ear therapy, drug delivery

## Abstract

Degeneration of neurons, such as the inner ear spiral ganglion neurons (SGN), may be decelerated or even stopped by neurotrophic factor treatment, such as brain-derived neurotrophic factor (BDNF), as well as electrical stimulation (ES). In a clinical setting, drug treatment of the SGN could start directly during implantation of a cochlear implant, whereas electrical stimulation begins days to weeks later. The present study was conducted to determine the effects of consecutive BDNF and ES treatments on SGN density and electrical responsiveness. An electrode drug delivery device was implanted in guinea pigs 3 weeks after deafening and five experimental groups were established: two groups received intracochlear infusion of artificial perilymph (AP) or BDNF; two groups were treated with AP respectively BDNF in addition to ES (AP + ES, BDNF + ES); and one group received BDNF from the day of implantation until day 34 followed by ES (BDNF ⇨ ES). Electrically evoked auditory brainstem responses were recorded. After one month of treatment, the tissue was harvested and the SGN density was assessed. The results show that consecutive treatment with BDNF and ES was as successful as the simultaneous combined treatment in terms of enhanced SGN density compared to the untreated contralateral side but not in regard to the numbers of protected cells.

## 1. Introduction

Cochlear implant technology is the state-of-the-art therapy for patients suffering from severe to profound sensorineural hearing loss. During surgery a silicone-based electrode device is inserted in the inner ear. Up to 22 electrode contacts electrically stimulate residual peripheral neurons of the hearing nerve and allow a hearing sensation for the deaf patients. Particularly at the electrode–nerve interface, there are pathophysiological processes occurring which can be manipulated by optimization of the biological aspects and combination of this approach with the implant. One of the most important problems which hinder further improvement of cochlear implant outcome is the secondary degeneration of the target cells of electrical stimulation, the spiral ganglion neurons (SGN) [1].

Sensorineural hearing loss is primarily caused by the loss of hair cells in the organ of Corti of the inner ear. Following hair cell loss, the auditory nerve degenerates. Initially, the afferent fibers of the SGN degenerate and subsequently the SGN and their central projections die [2,3,4].

Electrical stimulation (ES) delivered by a cochlear implant may provide trophic input to the neural structures of the inner ear and studies suggest that ES can preserve SGN following deafness [5,6,7]. However, results concerning the protective effects evoked by ES alone are inconsistent across research groups [8,9,10]. An additional strategy to enhance SGN survival following deafness is to replace the lost endogenous neurotrophic factor (NTF) support through local drug delivery [11,12,13] and thus prevent degeneration.

The combination of electrical stimulation with growth factor treatment presents a scenario closer to the normal clinical situation in which the auditory nerve is actively depolarized by the cochlear implant and trophic factors can support the neuronal survival. An additive effect on enhancing survival of SGN was found with the combined application of electrical stimulation and NTF such as BDNF [14,15] and glial cell line-derived neurotrophic factor (GDNF) [16,17]. Since candidates for cochlear implantation typically seek therapy following some period of deafness, an animal model with a delay lasting a few weeks between deafening and start of treatment is closer to the clinical situation than treatment initiation immediately post deafening. In a guinea pig model, a reduction in SGN numbers to 60% of normal can be observed after 4 weeks, whereas after 2 weeks no significant reduction was detected [18]. Other authors reported a reduced SGN survival after 2 weeks [1]. Therefore, several studies used a delay of 3 weeks for their investigations [17,19]. Previous studies assessed the survival of spiral ganglion cells after a period up to six weeks following deafness before treatment with BDNF plus fibroblast growth factor 1 (FGF1) [19] or before combined treatment with GDNF and ES [17] or BDNF and ES [20]. The results of these studies confirmed that NTF treatment benefits can be significantly increased by the additional application of electrical stimulation even with a treatment delay after onset of deafness. Furthermore, when ES and BDNF were applied simultaneously but ES was delivered 2 to 6 weeks longer than BDNF, the positive effects of the combined treatment were maintained in a guinea pig study [21]. Additionally in a study with deafened kittens, ES alone was able to preserve the positive effects of a previous combined treatment [22]. As the electrical stimulation of cochlear implant patients starts days or weeks after implant surgery while treatment with NTF could potentially start directly with implantation, a consecutive treatment of the patients with NTF and ES might be closest to clinical practice. The aim of the current study was to investigate the effect of a delayed consecutive treatment on SGN density and electrical responsiveness and compare it with the effects of BDNF or ES treatment alone or in the combined condition.

## 2. Materials and Methods

### 2.1. Experimental Subjects

Thirty-five healthy pigmented guinea pigs of both gender (Charles River WIGA GmbH, Sulzfeld, Germany), weighing between 280 and 550 g (age at implantation between 4 and 8 weeks), were used in the study in accordance to the German “Law on Protecting Animals” and to the European Communities Council Directive 86/609/EEC for the protection of animals used for experimental purposes. All experiments were approved by the Institutional Animal Care and Research Advisory Committee and permitted by the local government (LAVES, registration no. 02/558 and 04/913).

All procedures were performed under general anesthesia with xylazine (10 mg/kg, i.m.; Rompun^®^, Bayer, Leverkusen, Germany) and ketamine (40 mg/kg, i.m.; Ketamin Gräub^®^, Albrecht, GmbH, Aulendorf, Germany).

**Figure 1:**
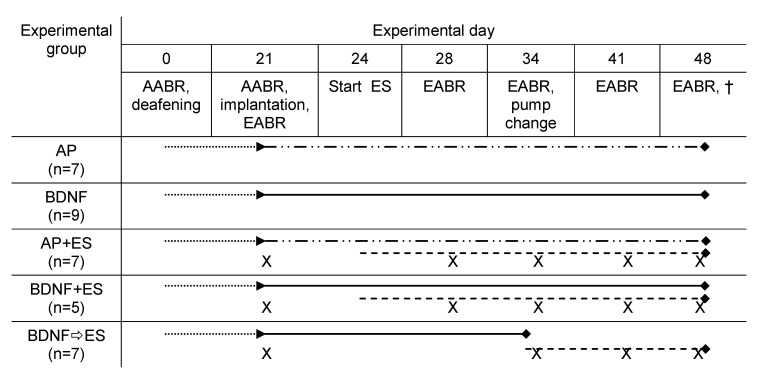
Experimental design. All animals were deafened on experimental day 0 after AABR measurement. After waiting for three weeks (dotted lines) deafening was verified by AABR measurements. Afterwards, an electrode pump array was implanted which delivered fluid to the inner ear (solid lines with dots: AP—artificial perilymph; solid line: BDNF—brain-derived neurotrophic factor, 50 ng/mL). The pumps were renewed on experimental day 34 in all subjects except of those of the BDNF ⇨ ES group. EABR measurements (indicated by “X”) were performed weekly in the stimulated groups (day 21, 28 (except group BDNF ⇨ ES), 34, 41 and 48). Electrical stimulation (ES) was performed in the groups as indicated by dashed lines.

The animals were divided into five treatment groups: artificial perilymph (AP; *n* = 7; control group), AP with chronic electrical stimulation (AP + ES; *n* = 7), BDNF alone (BDNF; *n* = 9), BDNF with chronic electrical stimulation (BDNF + ES; *n* = 5) and, as fifth group, BDNF treatment from experimental day 21 until day 34 followed by ES from day 34 until day 48 (BDNF ⇨ ES; *n* = 7). An overview of the different treatment groups is provided in Figure 1.

### 2.2. Electrode Drug Delivery Device

**Figure 2:**
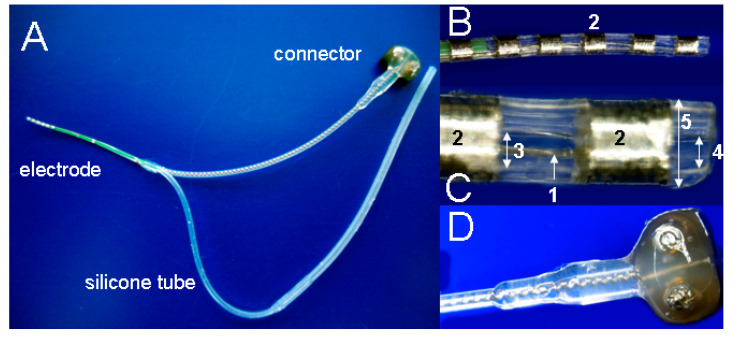
(**A**) The implant consists of an intracochlear electrode array and a silicone tube for local drug delivery. (**B**) Tip of the electrode with 6 contacts (2). (**C**) Further magnification of the electrode tip to visualize the platinum–iridium wires (1), the contacts (2), the drug delivery channel (3) and its opening (4) in the silicone matrix (5). (**D**) Ventral view of the connector.

The device (Cochlear Ltd., Sydney, Australia) consisted of six platinum contacts, 0.3 mm each, with 0.4 mm spacing (Figure 2). Beginning on the electrode tip, only the first and fourth contacts were linked to the connector via parylene-insulated platinum–iridium wires. The wires were embedded in a silicone matrix of about 450 µm outer diameter. The silicone matrix included a drug delivery channel (diameter: ~200 µm) with a single opening at the electrode tip. Wires and the drug channel separated in a distance of 22 mm from the tip (Figure 2A). During surgery, the silicone tube for drug delivery was connected to the flow moderator of a mini-osmotic pump with an infusion rate of 0.5 µL/h, suitable for 14 days delivery (Alzet model 2002; Durect Corp., Cupertino, CA, USA). The day before surgery, the pumps were either filled with AP with addition of 0.1% guinea pig serum albumin (Sigma-Aldrich, Steinheim, Germany) [19] or 50 ng BDNF (R&D Systems, Wiesbaden, Germany) diluted in 1 mL of serum albumin containing AP and primed in saline at 37 °C overnight.

### 2.3. Acoustically Evoked Auditory Brainstem Response (AABR)

Acoustically evoked auditory brainstem response (AABR) measurements verified normal hearing bilaterally in all tested animals prior to inclusion in this study. The hearing threshold of each subject was evaluated as previously described [17]. In brief, on experimental days 0 and 21, click stimuli were delivered to anesthetized animals and the neural responses were recorded, filtered (between 0.1 and 3 kHz) and averaged. The hearing threshold, defined as the lowest stimulus level that generated a visually replicable waveform in normal hearing guinea pigs, was determined. A minimum threshold shift of at least 50 dB SPL was required for deafened animals on day 21 to be included in this study.

### 2.4. Deafening Procedure

After day 0 AABR measurements all animals were deafened under general anesthesia by application of kanamycin and ethacrynic acid. Kanamycin was applied subcutaneously (400 mg/kg) and two hours later 40 mg/kg ethacrynic acid was injected into the jugular vein of the animal. The method was adopted from West [23].

### 2.5. Surgical Implantation Procedure of the Electrode and the Pump

On experimental day 21, after confirmation of deafness by AABR measurement, all animals were implanted with the electrode drug delivery device (Figure 2). The electrode was implanted unilaterally in the scala tympani of the left inner ear via the round window; the drug delivery channel was filled with the solution to be delivered, connected to the mini-osmotic pump; and the pump was positioned between the scapulae as previously described [17]. Epidural recording electrodes and restraint bolt holding screws were implanted as described by [5]. Prior to the sealing of the bulla defect with carboxylate cement (Durelon^®^, ESPE Dental AG, Seefeld, Germany), electrically evoked auditory brainstem responses (EABRs) were recorded to confirm functionality of the electrode array.

### 2.6. Electrically Evoked Auditory Brainstem Response (EABR)

Electrically evoked ABRs were recorded on experimental day 21 directly after electrode implantation and subsequently on days 34, 41 and 48 of the experiment in all groups with ES (AP + ES, BDNF + ES, BDNF ⇨ ES) and on day 28 in groups AP + ES and BDNF + ES. Monophasic current pulses (duration: 50 µs) were presented at 50 Hz through a 10 MHz Pulse Generator (TGP 110 10 MHz Pulse Generator, Thurlby Thandar instruments, Huntingdon, UK). With a custom-made converter, every second stimulus was changed to negative phase thus creating alternating pulses. Responses were recorded according to Mitchell et al. (1997) with electrodes placed at the vertex (1 cm posterior to bregma) and midline (2 cm anterior to bregma), and 1 cm lateral to bregma, ipsilateral to the implant (ground electrode). The averaged response to 500 presentations of a given stimulus was recorded using a Vicking IV device (Nicolet Biomedical Corp., Madison, WI, USA). The stimulus level was adjusted in steps of 10 µA. The threshold was defined as the lowest stimulus level that evoked a 1 µV or larger replicable wave III.

### 2.7. Impedance Measurement

Impedances were measured after each EABR measurement using a current of 100 µA. After a series of measurements with known resistances between 2.19 kΩ and 14.93 kΩ, a linear calibration curve was established that allowed the transformation of voltage values provided by an oscilloscope into impedance values.

### 2.8. Chronic Electrical Stimulation

Animals of the BDNF + ES and AP + ES group received continuous pulsatile electrical stimulation for 24 h a day for 24 days beginning on day 24 (3 days after implantation) via a portable electrical stimulator being mounted on the head of the animal (provided by the University of Michigan, Ann Arbor, MI, USA). Electrical stimulation presented biphasic charge-balanced pulses (100 µs per phase, 250 Hz at 40% duty cycle) 8 dB above the electrical response threshold. The design of the electrical stimulator has been described in Mitchell et al. (1998) in more detail. The BDNF ⇨ ES group was electrically stimulated accordingly from day 34 to day 48 after stopping delivery of BDNF on day 34.

### 2.9. Histological Procedures

On experimental day 48, 200 mL phosphate buffered saline (PBS) followed by 200 mL of 4% glutardialdehyde in PBS were perfused transcardially under general anesthesia. The temporal bones were isolated from the skull base and the bulla was opened and examined for tissue reactions and infections. The electrode was extracted and the cochlea was prepared for histology following the previously described procedures for fixation and decalcification of guinea pig cochleae [17]. The tissue was embedded in paraffin, serially sectioned at 5 µm and mounted on glass slides in order to quantitatively assess the number of SGN. Midmodiolar sections were chosen for evaluation as these provide six to seven cross sections of the Rosenthal’s canal. Images were taken, SGN were counted and the profiles of each Rosenthal’s canal were assessed. Only neurons with a minimum perikaryal diameter of 12 µm and a discernible nucleus were chosen and included for analysis. This approach led to a parameter called the SGN density, referring to the number of SGN per 10,000 µm^2^ [24]. Since SGN counting and area measurements were not always reliable at the most apical sites, these measurements were combined with the fourth middle turn.

Measurements and quantification were performed microscopically at a magnification of 200× (Olympus CKX41, Hamburg, Germany). Images were taken with a charge-coupled device (CCD) camera (colorview XS, SIS, Muenster, Germany) and processed using an image analysis program (analySIS Version. 3.2, Olympus, Hamburg, Germany).

### 2.10. Data Analysis

All data of SGN density as well as EABR threshold were tested for normality and are reported as mean ± SD for descriptive statistics. The paired t-test was applied to analyze the SGN density differences between the treated left side and the untreated right side within the animals of one group as well as for the analysis of SGN density differences between the basal and apical cochlear turns within one group. Group comparison was conducted depending on the result of the normality test, either by using ANOVA followed by Tukey’s multiple comparison test or by using Kruskal–Wallis test followed by Dunn’s post-test.

For comparison of EABR thresholds at different time points within one group, repeated measured ANOVA followed by Dunnett’s multiple comparison or, in the case of non-Gaussian distribution, the Friedman test followed by Dunn’s post-test were applied. All analyses were performed using GraphPad Prism software (La Jolla, CA, USA).

## 3. Results

### 3.1. AABR and Deafening

The animals had an initial hearing threshold of 34 ± 5 dB SPL (mean ± SD). The kanamycin and ethacrynic acid treatment resulted in all cases in an AABR threshold shift of at least 50 dB (average: 75 ± 9 dB).

### 3.2. Functional Results Based on EABR Measurements

**Figure 3:**
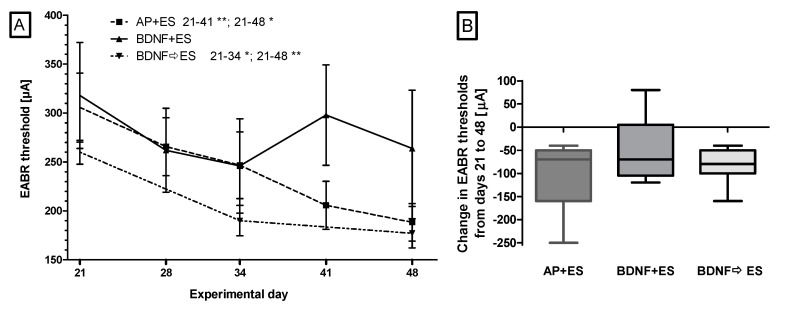
Electrically evoked auditory brainstem responses (EABR) were recorded weekly in all experimental groups except the AP group after electrode implantation from days 21 to 48 (exception: group BDNF ⇨ ES: no measurement on day 28). (**A**) depicts the mean ± SD EABR hearing thresholds over time for each electrically stimulated group. A reduction in threshold was detected only in groups AP + ES and BDNF ⇨ ES. (**B**): No differences between groups were found when comparing the mean threshold shifts between days 21 and 48. * *p* < 0.05; ** *p* < 0.01.

To monitor treatment related threshold changes, electrically evoked auditory brainstem responses were recorded on day 21 and weekly throughout the experiment in all electrically stimulated animals except for animals in group BDNF ⇨ ES, where no measurements were performed on day 28. The development of the average EABR hearing threshold of all electrically stimulated groups is plotted in Figure 3A. The EABR threshold (mean ± SD) of the BDNF ⇨ ES and AP + ES groups decreased significantly during the treatment period from 260 ± 32 µA and 305 ± 93 µA right after implantation to 177 ± 39 µA (*p* < 0.05) and 202 ± 57 µA (*p* < 0.01) on day 48, respectively. In animals with BDNF and simultaneous chronic electrical stimulation (BDNF + ES), the highest thresholds were also measured on day 21 (318 ± 54 µA) but the lowest on day 34 (246 ± 48 µA), whereupon the average threshold increased again by day 41, resulting in a mean threshold of 264 ± 133 µA at day 48. In this group, no significant differences between experimental days were detected. Comparing the mean threshold shifts between days 21 and 48 of the three stimulated groups, no significant differences between the groups were observed (Figure 3B).

**Figure 4:**
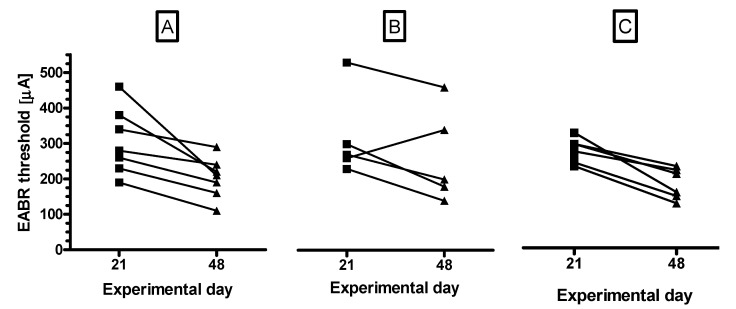
Threshold shifts between days 21 and 48 for groups AP + ES (**A**), BDNF + ES (**B**), and BDNF ⇨ ES (**C**). Provided are the individual developments of the hearing thresholds for all animals. The hearing threshold decreased in all electrically stimulated animals except one in the BDNF + ES group (**B**).

A more detailed overview on the hearing threshold changes for all electrically stimulated animals between days 21 and 48 is provided in Figure 4. Only in one case in the BDNF + ES group, the hearing threshold increased during the experimental period (Figure 4B). In all other cases the threshold decreased.

### 3.3. Impedance Measurements

At the time of EABR measurements, electrode impedances were also measured. Impedances increased between implantation on day 21 and day 48 by 3.8 ± 3.0 kΩ in the AP + ES group, by 2.7 ± 1.6 kΩ in the BDNF + ES group, and by 3.8 ± 2.7 kΩ in the BDNF group with consecutive ES. No differences between groups were detected (data not shown).

### 3.4. Histological Results

#### 3.4.1. Spiral Ganglion Cell Survival within Each Group

**Figure 5:**
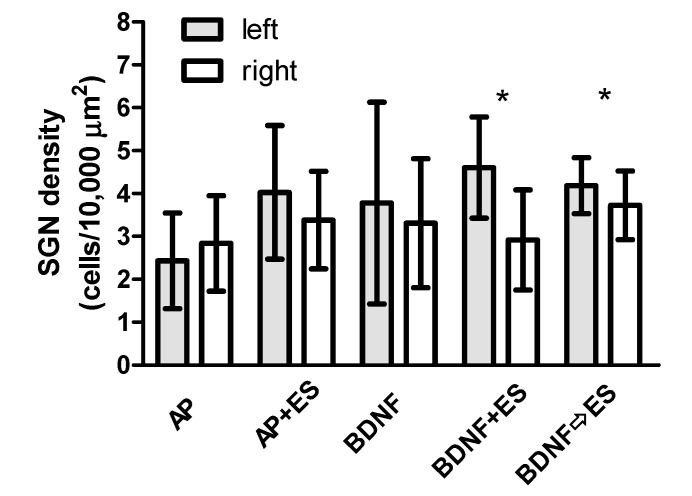
Density of surviving neurons (number of neurons per 10,000 µm^2^) for treated (left) and untreated (right) cochleae (mean ± SD). The density is increased on the treated side only in both groups receiving BDNF and ES. No differences were found when comparing the treated sides of all groups. * *p* < 0.05.

All subjects used in this study were implanted in the left cochlea, and the contralateral ear served as internal control. Figure 5 shows the comparison between the mean SGN density of the untreated right ears and the treated left ears within each group. The implanted cochleae of all groups except the AP group showed higher SGN densities than the untreated contralateral ears, even though this difference was significant only in BDNF treated groups with simultaneous or consecutive electrical stimulation (*p* < 0.05 in both cases).

Comparing the treated cochleae of all experimental groups as well as comparing all BDNF and or ES treated ears with the AP-treated control ears, no differences in the mean SGN density were observed. The mean density of SGN was lowest in AP-treated animals (2.4 ± 1.1 SGN/10,000 µm^2^). In the other groups, SGN density on day 48 was between 3.8 ± 3.3 SGN/10,000 µm^2^ (BDNF) and 4.6 ± 1.2 SGN/10,000 µm^2^ (BDNF + ES) with relatively large variations between animals.

#### 3.4.2. Protected Spiral Ganglion Cells

**Figure 6:**
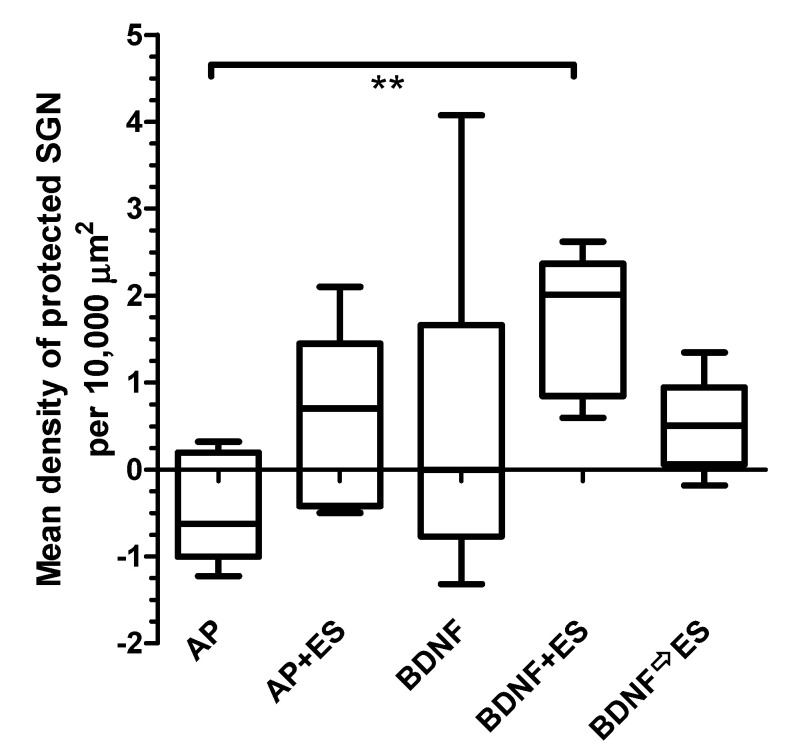
Median as well as minimal and maximal values of the density of protected SGN (density of neurons on the treated side minus density on the untreated side). Only in the BDNF + ES group, the density of protected neurons is increased when compared to the implant-only and AP-treated group. ** *p* < 0.01.

To evaluate the density of protected SGN per animal, the differences between the densities of surviving SGN in the implanted and treated cochleae and the non-implanted contralateral cochleae were calculated. The mean densities of protected SGN of all four treatment groups (Figure 6) were compared to the AP group (0.5 ± 0.6 SGN/10,000 µm^2^), and neither chronic electrical stimulation nor BDNF treatment alone had a protective effect on SGN survival (0.6 ± 0.9 and 0.5 ± 1.9 SGN/10,000 µm^2^, respectively). The protection of SGN achieved by the combined treatment with BDNF and ES was statistically significant (*p* < 0.01) when compared to the AP group (1.7 ± 0.8 SGN/10,000 µm^2^). Delivery of BDNF and consecutive ES resulted in 0.5 ± 0.5 protected SGN/10,000 µm^2^ and was not different when compared to the AP group. No differences in the density of protected SGN were observed between the AP + ES, BDNF, BDNF + ES and BDNF ⇨ ES groups. Variability in results was lowest in both groups receiving BDNF and ES.

#### 3.4.3. Comparison between Basal and Apical Turns

**Figure 7:**
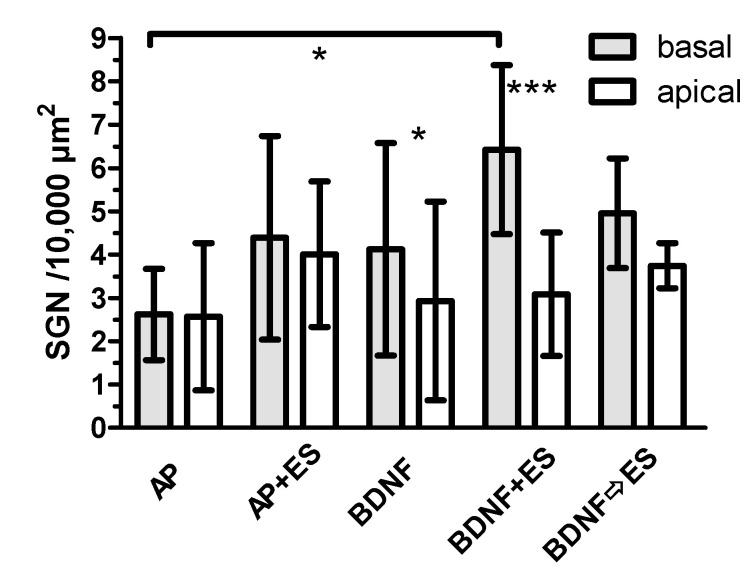
Comparison of the SGN density in basal and apical turns (mean ± SD). In BDNF and BDNF + ES groups, this difference was significant (asterisks above the respective bars). The number of SGN in the basal turn was increased compared to the AP group only in the BDNF + ES group, as indicated at the horizontal line. * *p* < 0.05; *** *p* < 0.001.

Comparing the numbers of SGN in basal (lower and upper basal) and apical (fourth middle and apical) turns, no differences were detected for AP, AP + ES and BDNF ⇨ ES groups. In the BDNF (*p* < 0.05) and BDNF + ES (*p* < 0.001) groups, SGN density was higher in the basal turn. SGN density was also significantly increased (*p* < 0.05) in the BDNF + ES group (6.4 ± 2.0 SGN/10,000 µm^2^) when compared to the AP group (2.5 ± 1.1 SGN/10,000 µm^2^) in basal turns (Figure 7).

## 4. Discussions

This study was conducted to examine functional and neuroanatomical effects of delayed BDNF treatment with consecutive chronic ES on the deafened guinea pig cochlea. The results demonstrate that chronic ES starting after cessation of the BDNF treatment is able to preserve increased SGN density compared to the untreated contralateral side (Figure 5). This is in line with results from earlier guinea pig [15] and cat [22] studies but goes also beyond the known findings, as both earlier studies applied ES and BDNF simultaneously before cessation of BDNF treatments and continuing with ES alone.

Previous in vitro and in vivo work indicates that BDNF can promote SGN survival [25,26,27,28,29]. In contrast, we did not detect a BDNF induced increase in SGN survival in the present study. This may be due to the relatively low BDNF concentration of 50 ng/mL used in the present study, which was chosen based on the bioefficacy study performed by Wefstaedt [29]. It cannot be excluded that this low concentration leads to a different activation of the BDNF receptors (tropomyosin-related kinase B (trkB) and p75NTR) and their following signal cascades, even though in vitro [29] and in vivo [30] beneficial effects of this BDNF concentration were reported. In contrast to the aforementioned studies where 50 ng/mL BDNF resulted in neuroprotection but was applied directly to cultured cells or 7 days after deafening in an animal model, cochlear implantation was delayed three weeks after deafening to induce SGN degeneration. Maybe with delayed treatment 3 weeks after deafening, the BDNF concentration of 50 ng/mL used in this study was too low for significant SGN protection in vivo.

ES did not significantly protect SGN from degeneration compared to the control group. Previous studies observed a SGN protective effect in vivo evoked by chronic electrical stimulation [5,7,31,32]. However, this effect is controversially discussed because other studies were not able to reproduce it [9,33,34]. One reason for the oppositional observations is that the beneficial effect of ES on neuronal survival depends on various parameters, such as pulse polarity, frequency, pulse width, healthiness of target cells and duration of stimulation. The underlying mechanism of the neurotrophic effect of depolarization is a sustained rise in cytosolic Ca^2+^, entering through L-type Ca^2+^ channels and the following intracellular biological cascade [35,36,37]. This effect of intracellular Ca^2+^ is in contrast to the critical role of cytosolic Ca^2+^ in mediating neuronal degeneration [38,39]. A hypothesis unifying these observations proposes that intracellular Ca^2+^ must rise to a particular “setpoint” to promote survival in the absence of neurotrophic factors; degeneration is the result of very high cytosolic Ca^2+^ [37,40,41]. Thus, survival occurs within a range of elevated Ca^2+^, with the lower end of the range determined by the Ca^2+^ setpoint for that neuron and the upper end by that neuron’s sensitivity to Ca^2+^-mediated neurotoxicity [41]. On the other hand, using exactly the same stimulus parameters, a protective effect of AP + ES was found earlier [17]. The only difference between both studies was that in the earlier study a monopolar stimulation paradigm was used, whereas in the current study, bipolar stimulation was used. As monopolar stimulation requires less current than the bipolar mode to reach threshold [42], this might explain the different results observed in our studies.

The combination of BDNF and ES resulted in a significant SGN protection. This confirms the results of other studies showing a synergistic effect of ES and BDNF regarding preservation of SGN (Shepherd et al., 2005). ES alone—when following a period of combined application with BDNF—was able to maintain the effect of the combined application for two to six weeks in a guinea pig model [21]. In our setting, ES alone started only after cessation of BDNF delivery but still resulted in an increased neuronal density compared to the untreated contralateral sides. This appears to be slightly surprising as both treatments alone did not evoke an effect. We can only speculate about possible reasons but provide four possible explanations. The first is that when starting ES, BDNF should still be available in the scala tympani from the previous delivery. This period might be short, but there could still be a synergistic effect. The second explanation may be that the subsequent ES, by upregulating the transcription of the BDNF gene, increases the secretion of endogenous BDNF [43,44,45] and therefore increases the maybe insufficient initial BDNF concentration of 50 ng/mL to a concentration which causes a biological effect. Additionally, high-frequency neuronal activity, induced by ES, can upregulate the number of BDNF-specific TrkB receptors on the surface of central nervous system neurons [46]. Due to the increased number of receptors, the ES could lead to an enhanced mode of action of the BDNF (remaining from the previous delivery or endogenously produced due to the ES) in the SGN. However, it should be noted that this effect is up to now not proven for peripheral neurons such as the SGN. The fourth explanation may be a statistical effect because, for some unknown reason, the standard deviation in the group with consecutive treatment is low compared to all other groups. In all other histological measures (number of protected cells and difference between basal and apical parts), the simultaneous BDNF and ES application resulted in an improvement, especially in the basal part, but not the consecutive treatment.

When evaluating the final EABR thresholds on day 48, no differences between AP + ES, BDNF + ES and BDNF ⇨ ES groups were detected. It has to be mentioned, however, that for AP + ES and BDNF ⇨ ES, a significant reduction in the threshold from implantation to day 48 was observed, whereas in the BDNF + ES group, there was only a tendency of reduced thresholds. The most likely reason for having not found a similar reduction in the BDNF + ES group, is the one animal with increasing thresholds from day 21 to 48. Impedance values of this animal were at 8.68 kΩ on day 48 and therefore only slightly higher than the mean value on this day in this group (6.4 kΩ).

A reduction over time in EABR thresholds was already reported by Kanzaki et al. (2002). They speculated that ES alone improves the functional state of SGN [16]. In contrast, Shepherd et al. (2005) measured a significant reduction in EABR thresholds only when BDNF was administered, either alone or in combination with ES [15]. Since we detected decreased thresholds in the AP + ES group, our data support the findings of Kanzaki et al. that ES reduces the EABR threshold over the treatment period.

There is a tendency of reduced SGN density in AP-treated ears compared to the respective contralateral ears. The effect may be due to increased neuronal degeneration due to the implantation, the delivery procedure, or the AP itself and is already reported in other studies that found lower SGN densities in deafened AP-treated cochleae when compared with the contralateral untreated deafened ears of the same animal [13,15].

Due to the relatively small sample sizes, the robustness of the statistical results could be limited but all together consecutive BDNF treatment and ES appears to be less effective in preventing neuronal degeneration than a combined application. As in a clinical setting ES typically starts a couple of days or weeks after implantation, a combined treatment right after implantation appears not to be realistic. A setup as used in a cat study [22] with an early start of BDNF delivery followed by simultaneous application of BDNF and ES for some time and prolonged ES after cessation of BDNF treatment might also be promising. It might be good to compare both approaches under controlled conditions.

## 5. Conclusions

Consecutive treatment with BDNF and ES was similarly effective as simultaneous treatment with BDNF and ES in terms of the density of surviving neurons when compared to the untreated contralateral side, but in all other measures the simultaneous treatment was more promising. Therefore, we conclude that under clinical conditions, both treatment strategies, BDNF application and electrical stimulation, should be combined. BDNF could be applied intraoperatively by use of a catheter or a drug depot for a longer release time followed by an early start of ES, as it is already implemented in some clinics worldwide, to ensure an early start followed by a sufficient period of time with a combined treatment.

## References

[1] Webster M., Webster D.B. (1981). Spiral ganglion neuron loss following organ of Corti loss: A quantitative study. Brain Res..

[2] Spoendlin H. (1971). Degeneration behaviour of the cochlear nerve. Arch. Klin. Exp. Ohren Nasen Kehlkopfheilkd.

[3] Ylikoski J. (1974). Correlative studies on the cochlear pathology and hearing loss in guinea-pigs after intoxication with ototoxic antibiotics. Acta Otolaryngol. Suppl..

[4] Dodson H.C., Mohuiddin A. (2000). Response of spiral ganglion neurones to cochlear hair cell destruction in the guinea pig. J. Neurocytol..

[5] Mitchell A., Miller J.M., Finger P.A., Heller J.W., Raphael Y., Altschuler R.A. (1997). Effects of chronic high-rate electrical stimulation on the cochlea and eighth nerve in the deafened guinea pig. Hear. Res..

[6] Miller J.M., Altschuler R.A. (1995). Effectiveness of different electrical stimulation conditions in preservation of spiral ganglion cells following deafness. Ann. Otol. Rhinol. Laryngol. Suppl..

[7] Hartshorn D.O., Miller J.M., Altschuler R.A. (1991). Protective effect of electrical stimulation in the deafened guinea pig cochlea. Otolaryngol. Head Neck Surg..

[8] Li L., Parkins C.W., Webster D.B. (1999). Does electrical stimulation of deaf cochleae prevent spiral ganglion degeneration?. Hear. Res..

[9] Agterberg M.J., Versnel H., de Groot J.C., van den Broek M., Klis S.F. (2010). Chronic electrical stimulation does not prevent spiral ganglion cell degeneration in deafened guinea pigs. Hear. Res..

[10] Chen I., Limb C.J., Ryugo D.K. (2010). The effect of cochlear-implant-mediated electrical stimulation on spiral ganglion cells in congenitally deaf white cats. J. Assoc. Res. Otolaryngol..

[11] Gillespie L.N., Shepherd R.K. (2005). Clinical application of neurotrophic factors: The potential for primary auditory neuron protection. Eur. J. Neurosci..

[12] Schwieger J., Hamm A., Gepp M.M., Schulz A., Hoffmann A., Lenarz T., Scheper V. (2020). Alginate-encapsulated brain-derived neurotrophic factor–overexpressing mesenchymal stem cells are a promising drug delivery system for protection of auditory neurons. J. Tissue Eng..

[13] Warnecke A., Scheper V., Buhr I., Wenzel G.I., Wissel K., Paasche G., Berkingali N., Jorgensen J.R., Lenarz T., Stover T. (2010). Artemin improves survival of spiral ganglion neurons in vivo and in vitro. Neuroreport.

[14] Chikar J.A., Colesa D.J., Swiderski D.L., Di Polo A., Raphael Y., Pfingst B.E. (2008). Over-expression of BDNF by adenovirus with concurrent electrical stimulation improves cochlear implant thresholds and survival of auditory neurons. Hear. Res..

[15] Shepherd R.K., Coco A., Epp S.B., Crook J.M. (2005). Chronic depolarization enhances the trophic effects of brain-derived neurotrophic factor in rescuing auditory neurons following a sensorineural hearing loss. J. Comp. Neurol..

[16] Kanzaki S., Stover T., Kawamoto K., Prieskorn D.M., Altschuler R.A., Miller J.M., Raphael Y. (2002). Glial cell line-derived neurotrophic factor and chronic electrical stimulation prevent VIII cranial nerve degeneration following denervation. J. Comp. Neurol..

[17] Scheper V., Paasche G., Miller J.M., Warnecke A., Berkingali N., Lenarz T., Stover T. (2009). Effects of delayed treatment with combined GDNF and continuous electrical stimulation on spiral ganglion cell survival in deafened guinea pigs. J. Neurosci. Res..

[18] Jyung R.W., Miller J.M., Cannon S.C. (1989). Evaluation of eighth nerve integrity by the electrically evoked middle latency response. Otolaryngol. Head Neck Surg..

[19] Miller J.M., Le Prell C.G., Prieskorn D.M., Wys N.L., Altschuler R.A. (2007). Delayed neurotrophin treatment following deafness rescues spiral ganglion cells from death and promotes regrowth of auditory nerve peripheral processes: Effects of brain-derived neurotrophic factor and fibroblast growth factor. J. Neurosci. Res..

[20] Song B.N., Li Y.X., Han D.M. (2008). Delayed electrical stimulation and BDNF application following induced deafness in rats. Acta Otolaryngol..

[21] Shepherd R.K., Coco A., Epp S.B. (2008). Neurotrophins and electrical stimulation for protection and repair of spiral ganglion neurons following sensorineural hearing loss. Hear. Res..

[22] Leake P.A., Stakhovskaya O., Hetherington A., Rebscher S.J., Bonham B. (2013). Effects of brain-derived neurotrophic factor (BDNF) and electrical stimulation on survival and function of cochlear spiral ganglion neurons in deafened, developing cats. J. Assoc. Res. Otolaryngol..

[23] West B.A., Brummett R.E., Himes D.L. (1973). Interaction of kanamycin and ethacrynic acid. Severe cochlear damage in guinea pigs. Arch. Otolaryngol..

[24] Meyer H., Stover T., Fouchet F., Bastiat G., Saulnier P., Baumer W., Lenarz T., Scheper V. (2012). Lipidic nanocapsule drug delivery: Neuronal protection for cochlear implant optimization. Int. J. Nanomed..

[25] Staecker H., Kopke R., Malgrange B., Lefebvre P., Van de Water T.R. (1996). NT-3 and/or BDNF therapy prevents loss of auditory neurons following loss of hair cells. Neuroreport.

[26] Gillespie L.N., Clark G.M., Bartlett P.F., Marzella P.L. (2003). BDNF-induced survival of auditory neurons in vivo: Cessation of treatment leads to accelerated loss of survival effects. J. Neurosci. Res..

[27] Nakaizumi T., Kawamoto K., Minoda R., Raphael Y. (2004). Adenovirus-mediated expression of brain-derived neurotrophic factor protects spiral ganglion neurons from ototoxic damage. Audiol. Neurootol..

[28] Shinohara T., Bredberg G., Ulfendahl M., Pyykko I., Olivius N.P., Kaksonen R., Lindstrom B., Altschuler R., Miller J.M. (2002). Neurotrophic factor intervention restores auditory function in deafened animals. Proc. Natl. Acad. Sci. USA.

[29] Wefstaedt P., Scheper V., Lenarz T., Stover T. (2005). Brain-derived neurotrophic factor/glial cell line-derived neurotrophic factor survival effects on auditory neurons are not limited by dexamethasone. Neuroreport.

[30] Miller J.M., Chi D.H., O’Keeffe L.J., Kruszka P., Raphael Y., Altschuler R.A. (1997). Neurotrophins can enhance spiral ganglion cell survival after inner hair cell loss. Int. J. Dev. Neurosci..

[31] Leake P.A., Hradek G.T., Snyder R.L. (1999). Chronic electrical stimulation by a cochlear implant promotes survival of spiral ganglion neurons after neonatal deafness. J. Comp. Neurol..

[32] Miller A.L., Prieskorn D.M., Altschuler R.A., Miller J.M. (2003). Mechanism of electrical stimulation-induced neuroprotection: Effects of verapamil on protection of primary auditory afferents. Brain Res..

[33] Araki S., Kawano A., Seldon L., Shepherd R.K., Funasaka S., Clark G.M. (1998). Effects of chronic electrical stimulation on spiral ganglion neuron survival and size in deafened kittens. Laryngoscope.

[34] Shepherd R.K., Matsushima J., Martin R.L., Clark G.M. (1994). Cochlear pathology following chronic electrical stimulation of the auditory nerve: II. Deafened kittens. Hear. Res..

[35] Gallo V., Kingsbury A., Balazs R., Jorgensen O.S. (1987). The role of depolarization in the survival and differentiation of cerebellar granule cells in culture. J. Neurosci..

[36] Koike T., Martin D.P., Johnson E.M. (1989). Role of Ca^2+^ channels in the ability of membrane depolarization to prevent neuronal death induced by trophic-factor deprivation: Evidence that levels of internal Ca^2+^ determine nerve growth factor dependence of sympathetic ganglion cells. Proc. Natl. Acad. Sci. USA.

[37] Collins F., Schmidt M.F., Guthrie P.B., Kater S.B. (1991). Sustained increase in intracellular calcium promotes neuronal survival. J. Neurosci..

[38] Choi D.W. (1988). Calcium-mediated neurotoxicity: Relationship to specific channel types and role in ischemic damage. Trends Neurosci..

[39] Kristian T., Ouyang Y., Siesjo B.K. (1996). Calcium-induced neuronal cell death in vivo and in vitro: Are the pathophysiologic mechanisms different?. Adv. Neurol..

[40] Koike T., Tanaka S. (1991). Evidence that nerve growth factor dependence of sympathetic neurons for survival in vitro may be determined by levels of cytoplasmic free Ca2+. Proc. Natl. Acad. Sci. USA.

[41] Hegarty J.L., Kay A.R., Green S.H. (1997). Trophic support of cultured spiral ganglion neurons by depolarization exceeds and is additive with that by neurotrophins or cAMP and requires elevation of [Ca2+] i within a set range. J. Neurosci..

[42] Zwolan T.A., Collins L.M., Wakefield G.H. (1997). Electrode discrimination and speech recognition in postlingually deafened adult cochlear implant subjects. J. Acoust. Soc. Am..

[43] Rocamora N., Welker E., Pascual M., Soriano E. (1996). Upregulation of BDNF mRNA expression in the barrel cortex of adult mice after sensory stimulation. J. Neurosci..

[44] Nanda S.A., Mack K.J. (2000). Seizures and sensory stimulation result in different patterns of brain derived neurotrophic factor protein expression in the barrel cortex and hippocampus. Brain Res. Mol. Brain Res..

[45] Balkowiec A., Katz D.M. (2000). Activity-dependent release of endogenous Brain-Derived Neurotrophic Factor from primary sensory neurons detected by ELISA in situ. J. Neurosci..

[46] Du J., Feng L., Yang F., Lu B. (2000). Activity- and Ca(2+)-dependent modulation of surface expression of brain-derived neurotrophic factor receptors in hippocampal neurons. J. Cell Biol..

